# Effects of sample age on data quality from targeted sequencing of museum specimens: what are we capturing in time?

**DOI:** 10.1186/s12864-020-6594-0

**Published:** 2020-02-28

**Authors:** Angela McGaughran

**Affiliations:** 1 Australian National University, Research School of Biology, Division of Ecology and Evolution, Acton, Canberra, ACT 2600 Australia; 2 CSIRO Land and Water, Integrated Omics Team, Black Mountain Laboratories, Canberra, ACT 2600 Australia

**Keywords:** Historical DNA, Library quality, Museum genomics, NGS, Targeted capture

## Abstract

**Background:**

Next generation sequencing (NGS) can recover DNA data from valuable extant and extinct museum specimens. However, archived or preserved DNA is difficult to sequence because of its fragmented, damaged nature, such that the most successful NGS methods for preserved specimens remain sub-optimal. Improving wet-lab protocols and comprehensively determining the effects of sample age on NGS library quality are therefore of vital importance. Here, I examine the relationship between sample age and several indicators of library quality following targeted NGS sequencing of ~ 1300 loci using 271 samples of pinned moth specimens (*Helicoverpa armigera*) ranging in age from 5 to 117 years.

**Results:**

I find that older samples have lower DNA concentrations following extraction and thus require a higher number of indexing PCR cycles during library preparation. When sequenced reads are aligned to a reference genome or to only the targeted region, older samples have a lower number of sequenced and mapped reads, lower mean coverage, and lower estimated library sizes, while the percentage of adapters in sequenced reads increases significantly as samples become older. Older samples also show the poorest capture success, with lower enrichment and a higher improved coverage anticipated from further sequencing.

**Conclusions:**

Sample age has significant, measurable impacts on the quality of NGS data following targeted enrichment. However, incorporating a uracil-removing enzyme into the blunt end-repair step during library preparation could help to repair DNA damage, and using a method that prevents adapter-dimer formation may result in improved data yields.

## Background

The technological innovations underlying next generation sequencing (NGS) have resulted in an unprecedented ability to obtain DNA sequence data from specimens encompassing the vast diversity of biological life [1–3]. In recent times, NGS has opened up possibilities not just for recovering DNA data from extant species, but also from historical samples and even extinct species. Collectively, this has shed light on human adaptation [4], relationships among humans and other hominids [5–7], and the place of extinct species, such as moa and mammoth, in evolutionary history [8, 9].

However, ancient DNA (aDNA; > 500 years old) has proven difficult to work with because of its fragmented nature – after the death of an organism, DNA is degraded by endogenous nucleases, as well as damaged by chemical and physical events [10]. In addition to short fragment length, aDNA is commonly characterised by an increased occurrence of purine residues before strand breaks [11], and an increased frequency of cytosine to thymine substitutions near the ends of fragments [12, 13]. These three features also appear in historical (i.e., hundreds vs. thousands of years old) samples [2, 14, 15], and those that have been subjected to harsh conditions (e.g., formalin fixation) during preservation [16]. Coupled with this, endogenous DNA is generally present in only small amounts in preserved specimens [17].

NGS methods compound these issues through loss of DNA in various steps of the library preparation protocol [18, 19]. Despite this, only a small number of studies have aimed at improving wet-lab protocols for NGS (e.g., [20–22]) and current conversion efficiencies remain around 30–70% [23–25]. Thus, there is scope for improving the efficiency of NGS methods through library preparation procedure manipulation.

In addition, the effects of sample age on NGS data quality are generally understudied in a quantitative framework. Though some studies have noted negative relationships between sample age and parameters such as read length and number of reads [3, 26–31], focused analyses of the ways in which sample age, alongside approaches employed during library preparation, may affect estimators of sequencing quality are rare. Such information is particularly pertinent to population-scale museum studies, where users would benefit from further understanding of the quantitative effects of sample age on sequencing quality and the adjustments to library preparation protocol that could improve sequence quality.

The first attempt at using temporal samples in a population-scale context was made nearly 30 years ago [32], and was followed by a suite of such studies harnessing the power of museum samples (reviewed in [33]). In more recent times, new genomic methods that can better cope with low concentrations of starting material have been developed. In particular, targeted enrichment has proven useful for working with degradation-vulnerable specimens because the bait sequences are short and the method involves an amplification step following hybridisation. As a result, users can obtain substantial amounts of sequence data despite working with low molarity, fragmented DNA [34–36]. Thus, since its first applications to museum samples in the early 2010s [2, 37–41], a January 2020 search on GoogleScholar indicates its now widespread use (search term ‘targeted enrichment museum’ brings up > 27,000 results, > 2200 of these are for 2019 only; https://scholar.google.com.au/scholar; accessed 17/01/2020).

Here, I use 271 pinned insect specimens of the pest moth, *Helicoverpa armigera*, to test the effects of sample age on NGS library quality following a targeted capture approach. I use a temporal gradient of samples (5 to 117 years) to compare the effects of different sample ages on several indicators of sequenced read quality and identify key areas in the library preparation protocol that users should consider carefully when planning their experiments.

## Results

### DNA damage analysis

**Fig. 1:**
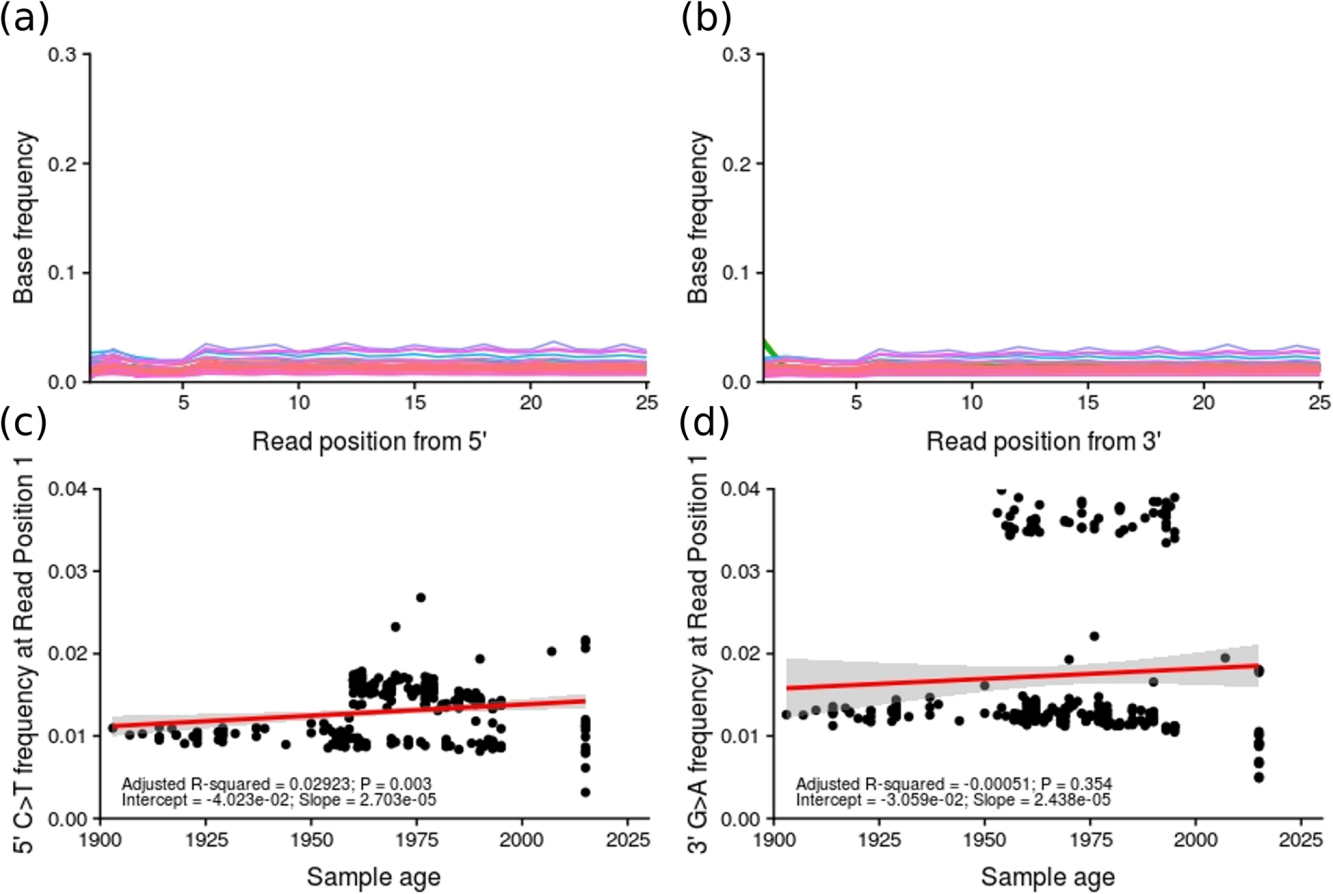
Plots of: **a** the frequency of C to T base substitutions from the 5′ end of each read; **b** the frequency of G to A base substitutions from the 3′ end of each read; **c** the frequency of C to T base substitutions at read position 1 from the 5′ end of reads vs. sample age; and **d** the frequency of G to A base substitutions at read position 1 from the 3′ end of reads vs. sample age. The expected signal for DNA damage due to age of specimens is an increased frequency of C to T and G to A transitions at the 5′ and 3′ end of reads, respectively, as well as a correlation between the frequency of such transitions at the 1st read position and sample age

The program, mapdamage [42], was used to assess and quantify the damage patterns associated with NGS of historical specimens. In particular, the frequency of cytosine to thymine (C → T) mutations from the 5′-ends, and guanine to adenine (G → A) mutations from the 3′-ends was examined, as these follow cytosine deamination; a common artefact in preserved DNA [2, 14, 43, 44]. I found no signal of C to T substitutions and G to A substitutions at high frequency at the 5′- and 3′-ends of mapped reads, respectively (Fig. 1).

### Relationship between sample age and NGS quality metrics

**Fig. 2:**
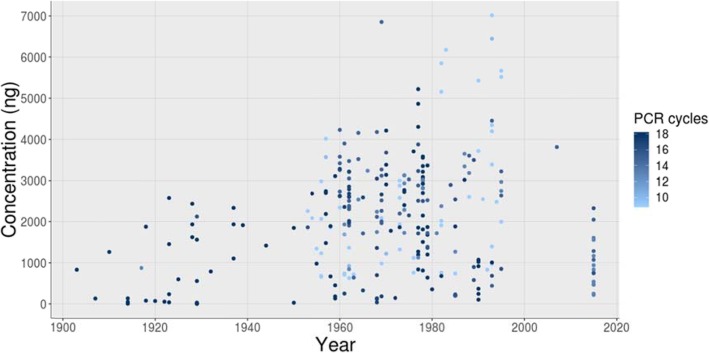
Plot demonstrating for each sample used in this study, the date of collection, the starting DNA concentration, and (via the colour scale of points, as indicated by the legend to the right) the number of PCR cycles required during the library preparation

I first checked to see whether older samples had lower starting material concentrations (i.e., lower concentrations after adapter fill-in), and therefore required a higher number of indexing PCR cycles during the library preparation procedures. Both effects were seen in the data (T_269_ = 3.83; *P <* 0.01; *R* = 0.23(0.11:0.34) and T_269_ = -5.56; *P < 0.01*; *R* = -0.32(− 0.42:-0.21), for starting concentration, and number of PCR cycles, respectively) and can be visualised in Fig. 2.

**Fig. 3:**
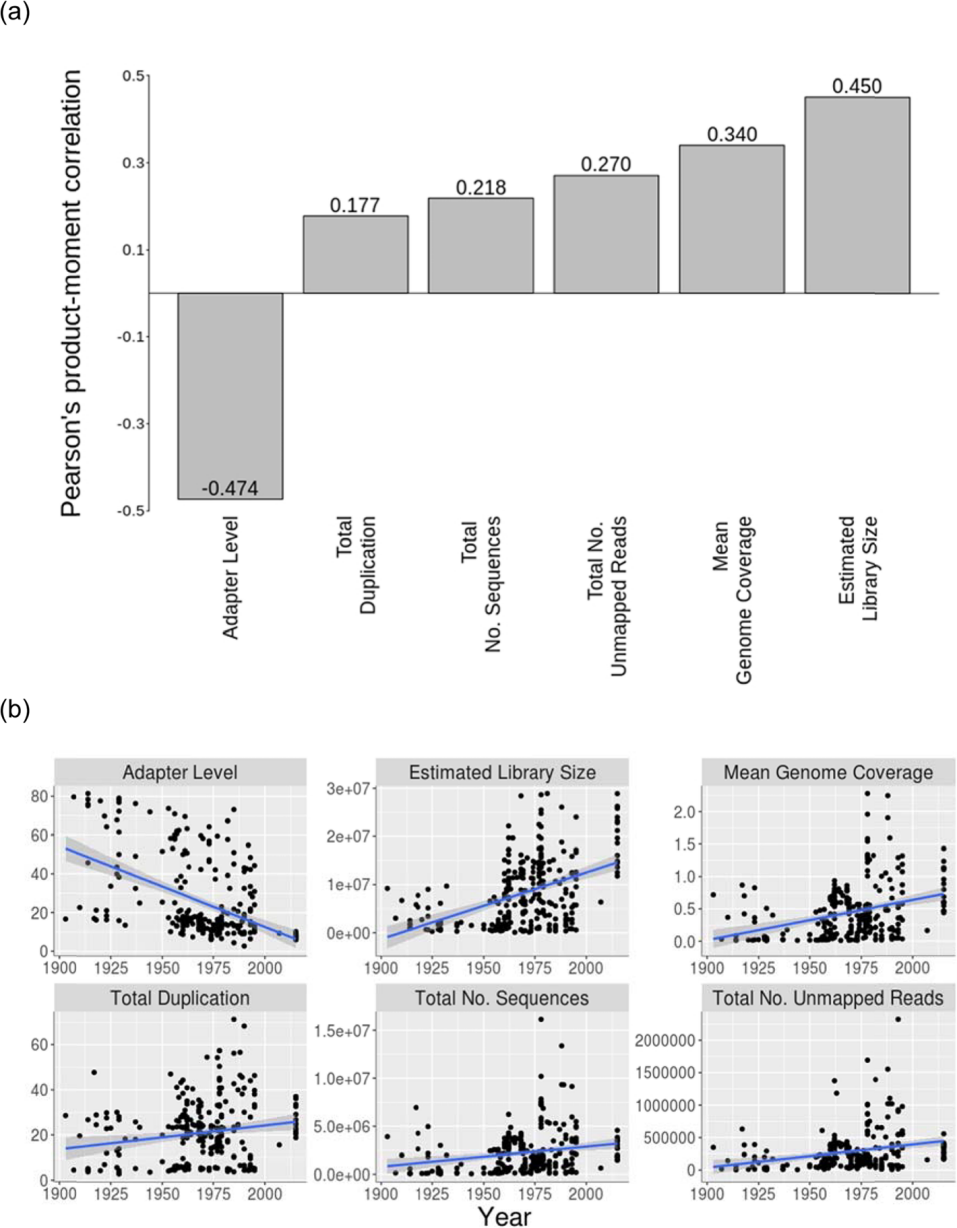
The correlation between sample age and various measures of sequencing quality, including adapter level, total duplication, total number of sequences, total number of unmapped reads, mean genome coverage, and estimated library size, when reads were aligned to the reference genome. In (**a**), each bar in the plot represents the strength and direction of correlation, and the R-value is displayed in/on the bars. In (**b**), the raw data points are plotted for each pair-wise comparison, along with the linear regression trend line. See Table 1 for the detailed statistics

Next, I examined the impact of sample age on several aspects of library quality, with respect to the alignment of reads to the *full* genome. Sample age was positively correlated with the total number of sequenced reads, the mean genome coverage, and the estimated library size (Fig. 3, Table 1). Meanwhile, as samples got older, the percentage of adapters increased significantly but, interestingly, the total percentage of duplication decreased slightly. The percentage of unmapped reads was not related to sample age (Fig. 3, Table 1).

**Table 1:** Correlation of sample age and several library quality metrics when reads were aligned to the full *Helicoverpa armigera* reference genome

Library quality metric	Correlation with sample age
Total number of sequences	T_269_ = 3.66; *P <* 0.01; *R* = 0.2180 (0.1015:0.3286)
Total duplication	T_269_ = 2.95; *P <* 0.01; *R* = 0.1771 (0.0592:0.2901)
Mean genome coverage	T_269_ = 5.93; *P* < 0.01; *R* = 0.34 (0.23:0.44)
Estimated library size	T_269_ = 8.24; *P <* 0.01; *R* = 0.45 (0.35:0.54)
Adapter level	T_269_ = -8.82; *P* < 0.01; *R* = -0.47(− 0.56:-0.38)
Total number of unmapped reads	T_269_ = 4.60; *P < 0.01*; *R* = 0.27 (0.16:0.38)

**Fig. 4:**
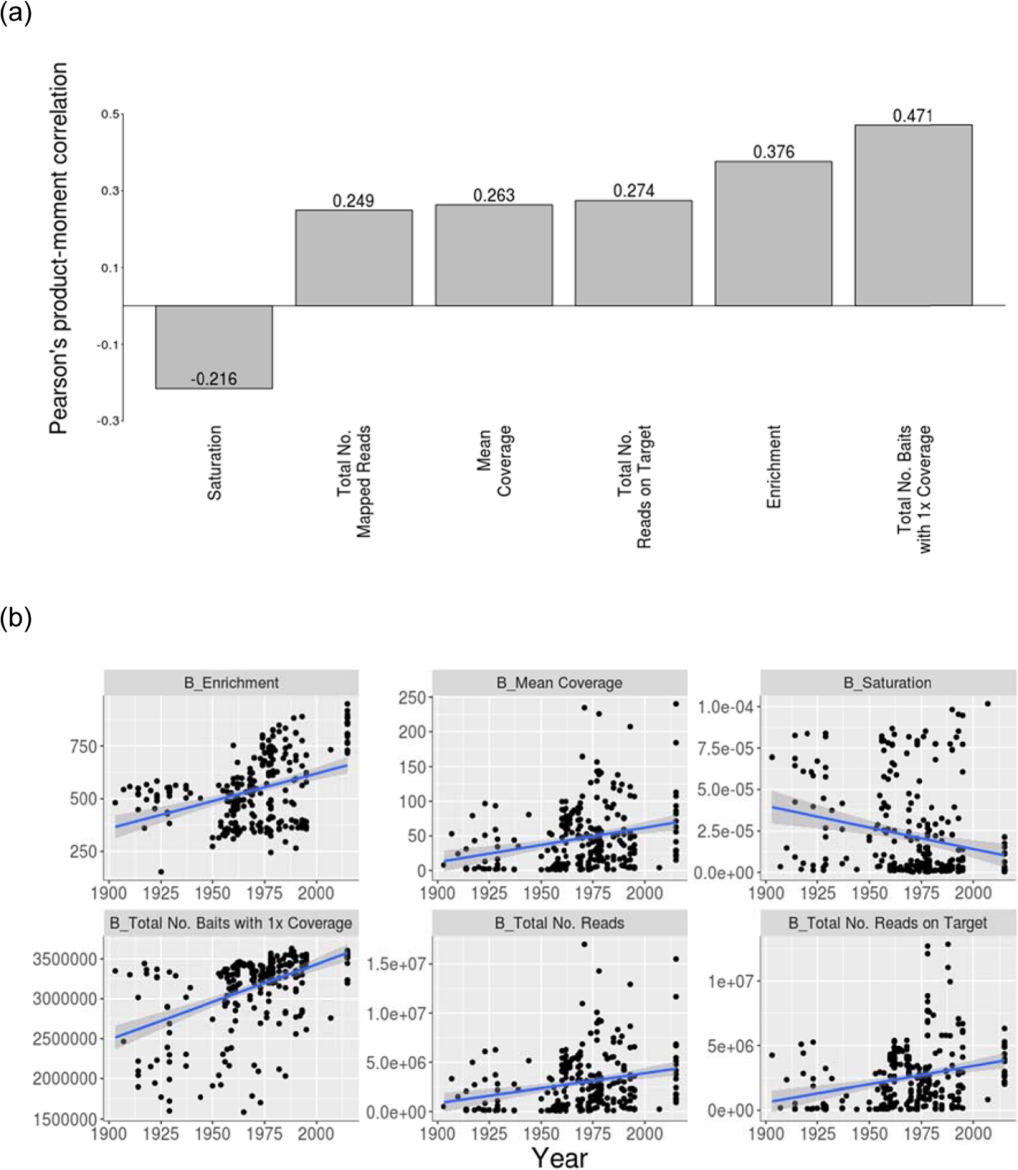
The correlation between sample age and various measures of sequencing quality, including saturation, total number of mapped reads, mean coverage, total number of reads on target, enrichment, and total number of baits with 1x coverage, when reads were aligned to the targets. In (**a**), each bar in the plot represents the strength and direction of correlation, and the R-value is displayed in/on the bars. In (**b**), the raw data points are plotted for each pair-wise comparison, along with the linear regression trend line. See Table 2 for the detailed statistics. Note that the total number of baits at 5x, 10x, 20x, and 30x coverage are excluded from these plots as they were highly correlated with the total number of baits at 1x coverage

To examine the effect of sample age on the success of the targeted capture, I next evaluated several metrics with respect to the mapping of reads against the targeted regions. Sample age was positively correlated with the percentage of reads that mapped to baits and the mean bait coverage, as well as the percentage of reads on target, and was highly correlated (R > 0.33; *P* < 0.01) with the percentage of baits covered from 1x to 30x (Fig. 4, Table 2). Meanwhile, the degree of saturation (which provides an indication of whether a higher sequence depth will translate into a higher percentage of covered positions) decreased as samples got older, as did the degree of enrichment (calculated as: on-target reads per Kb/off-target reads per Kb) (Fig. 4, Table 2).

**Table 2:** Correlation of sample age and several library quality metrics when reads were aligned to the targeted regions from the *Helicoverpa armigera* reference genome

Library quality metric	Correlation with sample age
Total number of reads	T_269_ = 4.38; *P <* 0.011.726e^−05^; *R* = 0.26 (0.14:0.37)
Mean coverage	T_269_ = 4.47; *P <* 0.01; *R* = 0.26 (0.15:0.37)
Total number of baits on target	T_269_ = 3.94; *P <* 0.01; *R* = 0.23 (0.12:0.34)
Total number of baits at 1x coverage	T_269_ = 8.77; *P <* 0.01; *R* = 0.47 (0.37:0.56)
Total number of baits at 5x coverage	T_269_ = 7.47; *P <* 0.01; *R* = 0.41 (0.31:0.51)
Total number of baits at 10x coverage	T_269_ = 6.95; *P* = *<* 0.01; *R* = 0.39 (0.28:0.49)
Total number of baits at 20x coverage	T_269_ = 6.31; *P <* 0.01; *R* = 0.36 (0.25:0.46)
Total number of baits at 30x coverage	T_269_ = 5.84; *P* = *<* 0.01 *R* = 0.34 (0.23:0.44)
Saturation	T_269_ = -0.22; *P <* 0.01; *R* = 0.22(−0.33:-0.10)
Enrichment	T_269_ = 6.65; *P* = *<* 0.01; *R* = 0.38 (0.27:0.47)

## Discussion

In this work, I showed that age of samples has a significant effect on data quality following targeted NGS in *H. armigera*. In particular, following mapping of reads all coverage-based metrics, both across the whole genome and across targeted regions, were significantly correlated with sample age, such that older samples showed poorer coverage when compared to younger samples. In addition, saturation (an indicator of whether additional sequencing would result in a higher capture coverage) decreased as samples got older; older samples are therefore less cost-efficient than younger samples in terms of per dollar sequencing output. Meanwhile, the degree of enrichment (a direct measure of targeted capture success) also decreased as samples got older. This is consistent with the coverage metrics, showing that older samples require more sequencing for a greater enrichment success.

Previous work has clearly shown a general pattern of DNA degradation over time. For example, a significant negative correlation between amounts of endogenous mitochondrial DNA and age has been shown in primates, horses, and cows [14], and a recent meta-analysis showed a bulk loss of DNA over time in samples of modern humans, herbarium plants, Columbian and wooly mammoths, horses, and polar bear [45]. The former study also found that fragment lengths did not decrease in a consistent manner over time [14]; instead they are hypothesised to rapidly reduce to a small average size following death before stabilising due to autolytic processes [14, 46].

Consistent with the current study for more recent (historic) DNA, research shows negative relationships between sample age and various measures of NGS read quality, including mean coverage, read length, missing data, and number of recovered loci [27, 29, 30]. For example, a 6% decrease in coverage of targeted regions with every 10 years of sample age and a lowering of mean read depth by 40x per 10 years was shown for formalin-fixed paraffin-embedded (FFPE) tissue samples up to 32 years old [26]. Meanwhile, a targeted capture experiment including 185 bird samples up to 142 years of age compared a subset of modern and historical samples for each of five bird species and also found significant negative relationships between sample age and the number of sequenced reads [31].

In the current study, mapdamage analysis detected no signature of deamination in the mapped reads. Previous work has shown that deamination, particularly the frequency of C → T substitutions, is common in old samples [13, 42, 45] and significantly positively correlated with age ([14, 47]; *R*^*2*^ = 0.45; *P* = 1.44 × 10^− 10^; *n* = 71 in the latter study). Generally, rates of both C → T and G → A substitutions towards the termini of reads tend to exceed 20% in samples > 500 years and can exceed 10% in historical samples [14], increasing towards 30% prior to soft-clipping [31].

The rate of hydrolytic deamination varies with temperature, pH, and salinity, thus different conditions during the original sample deposition or capture are likely to account for any differences among samples [14]. Though much of the preservation journey of the samples studied here is unknown, the moths were all field-caught and preserved as pinned specimens and, based on techniques in use in recent history, it is likely they were killed using ethyl acetate, which has been shown to produce degraded, low molecular weight DNA [48, 49]. Analysis of the moth DNA extracts confirmed a high level of degradation (most fragments < 500 bp), and research suggests that samples treated with harsh preservation methods (e.g., ethyl acetate, bleach), are likely to have an accelerated rate of deamination [14]. As a result, there is a reasonable likelihood that the moth DNA used here had been subject to chemical, as well as time-based, degradation. Such signals are unlikely to have been masked by contamination in the current study, because assessments of damage were made using sequence reads that had already been aligned to the reference genome. In addition, the percentage of unmapped reads was not related to sample age, indicating that contamination does not correlate with the age of the sample. Though I cannot be absolutely certain, this suggests that USER enzyme (Uracil Specific Excision Reagent, which functions to remove uracil residues and repair resulting abasic sites; see Methods), was effective in the current study, and its role in repairing DNA damage is well-supported in the literature [13, 50–52].

This is particularly true for ancient DNA samples, where comparisons of samples with and without uracil-removal treatment have shown marked reductions in common signals of DNA damage. For example, in one study of 11 cave bear bones (25,000–50,000 years old), a comparison of DNA molecules from 87 treated clonal samples showed zero G/C → A/T substitutions, while 19 such substitutions were present in the 79 clones that received no uracil-removal treatment [51]. Marked reductions in deamination damage patterns have been similarly shown for historical studies. For example, Gorden et al. [53] compared untreated and treated forensic bone samples up to 50 years of age and found G/C → A/T substitutions of 3–15 and < 1%, respectively. Meanwhile, Bi et al. [2] used an enzyme which stalls amplification of templates containing uracil and found C → T frequencies of ~ 0.6%. In contrast, studies of similarly-aged (~ 100 years) museum samples that used no uracil-based treatment display C → T transition rates nearly an order of magnitude higher (2–4% [14];), and as high as 30% in some cases ([31]; see above). In the current study, the maximum rate of C → T transitions at the first position in mapped 5′ reads was ~ 0.04%.

The benefit of removing deaminated sites is that, left intact, they can lead to sequencing errors, particularly in low-coverage sequencing experiments [13]. However, there is a potential trade-off to consider. Uracil removal will cut all of the affected DNA fragments, thus potentially resulting in samples in which the majority of fragments have been cut [13, 51]. Even if employing a method to repair the DNA fragment after cutting (see Methods), if the starting DNA is highly degraded, the post-USER fragments could be too short to generate a final library of meaningful length. Ultimately, the recommendation to use a uracil-removing or stalling enzyme should come down to an understanding of the level of fragmentation, the preservation method, and the likelihood of deamination, of ones’ samples. An alternative to such treatment is the removal of false variant calls bioinformatically, e.g., by trimming the ends of reads; however this may lead to a high loss of data if done conservatively.

In the current study, the percentage of adapter contamination was much higher in older samples (up to ~ 82% in the oldest samples), which tended to have lower starting concentrations and therefore usually required a higher number of indexing PCR cycles. Adapter dimers form when the adapters self-ligate instead of ligating to the sample DNA, and such dimers can dominate during PCR, which has a tendency to amplify shorter fragments more efficiently than longer ones [54]. In addition, adapter dimers form clusters at high efficiency and therefore consume valuable flow cell space during sequencing, resulting in a high proportion of wasteful adapters in the sequenced reads [55], as found here. Adapter concentrations can be optimised prior to library preparation and dimers can usually be removed by doing some form of bead or gel-based clean-up or titration [56], but, in the case of old and fragmented samples, the adapter-dimer is often very similar in size to the ligated library (~ 120 cf ~ 150 bp) and can therefore be very difficult to remove [24, 57]. Fortunately, new kit-based methods can prevent adapter-dimer formation during library preparation (e.g., DimerFree technology from Tecan Genomics Inc., or Dimerator™ technology from DiaCarter, which blocks PCR amplification of adapter-adapter products). Though such methods were not widely available at the time the lab work for this project was undertaken, I have since used the UltraLow Ovation Kit (Tecan Genomics Inc.) on historical moth specimens of the same species and enjoyed significantly reduced adapter-dimer levels (< 5%).

## Conclusions

Based on the findings presented here and, as shown previously, museum samples are a great resource for answering an array of evolutionary questions, but there are inherent challenges linked to DNA degradation. Here, I identify two major considerations users should carefully consider when following standard library preparation protocols during targeted capture experiments of historical samples. First, after careful consideration of the likelihood of sample deamination, USER enzyme or similar incorporated into the blunt end-repair step could be a good option for removing and repairing DNA damage associated with historical specimens. Second, adapter contamination can be extremely high in sequence reads of older samples, thus users may find it particularly helpful to consider a method that guarantees prevention of adapter-dimer formation. Each of these considerations may result in improved yields and reduced DNA damage in the sequenced reads, thus improving final data quality.

Over the past two centuries, museum collections have grown in size and importance [58, 59] and simultaneous advances in sequencing technologies have unleashed a new frontier in museum genomics [2, 60, 61]. Indeed, museums hold indispensable records of the past, and act as libraries of biological diversity in time and space. As wet-lab protocols, sequencing methods, and bioinformatic pipelines continue to improve and evolve, ancient and archival DNA samples will become even more valuable resources for the study of diverse historical processes.

## Methods

### Sample selection

A total of 271 pinned specimens of the insect pest moth, *Helicoverpa armigera*, were obtained from several museums and/or government departments across Australia, including the Australian National Insect Collection (Canberra), the Department of Agriculture and Food (Western Australia), the Department of Agriculture and Fisheries (Queensland), the Agricultural Scientific Collections Trust (New South Wales), and Museum Victoria (Victoria); specimens were collected at various time points between 1903 and 2015 (Fig. 1).

### Genomic DNA preparation

A ‘salting-out’ protocol [62] was followed to extract genomic DNA for all pinned specimens of *H. armigera*. Though described more than 20 years ago, this method is still highly used and has recently been shown to produce higher DNA yields than other extraction methods, including phenol chloroform and kit-based [63]. I also found that the more expensive kit-based extraction methods gave equivalent starting concentrations to salting-out in a small test (data not shown). Here, the salting-out protocol was followed with a modification to the first step, which involved soaking moth abdomens in buffer with Proteinase K (#19133, Qiagen) for 24 h. A salt solution was then added to the abdomen preparation (after the abdomen was removed and cleaned), as per the recommended protocol [62].

### Library preparation

Library preparation was broadly based on the procedures outlined in [20], using the standard steps for NGS library preparation (i.e., end repair, adaptor ligation and fill-in, and indexing PCR), but with several modifications due to the fragmented nature of the starting material. These modifications included: (1) the omission of a shearing step: (2) the incorporation of Uracil-Specific Excision Reagent (USER) enzyme (#M5508, New England Biolabs, Inc.) in the blunt-end repair step; (3) an ‘on-beads’ clean-up protocol throughout (thus, no elution was performed following the majority of bead clean-ups); and (4) the use of a calculation to determine the number of required indexing PCR cycles.

I omitted the shearing step because sample aliquots (3 μl) run on a 1% agarose gel following DNA extraction showed DNA to predominantly be < 500 bp in length for all samples. USER was included because deamination is a recognised outcome of DNA degradation processes in historical samples and the enzyme functions to excise uracil sites, forming an abasic (apyrimidinic) site while leaving the phosphodiester backbone intact. When followed by T4 DNA polymerase (#EP0062, ThermoFisher Scientific) treatment (a standard step in library preparation), the result is removal of uracil residues from the DNA, cleavage of the 5′- and 3′- sides of the resulting abasic sites, and removal of the 3′-phosphate groups by T4 polynucleotide kinase (PNK; EK0032, ThermoFisher Scientific). Thus, USER removes the DNA damage, but the treated molecules are repaired and retained in the library. The on-beads protocol (SeraMag beads; #45152105050250, GE Healthcare Life Sciences) involved carrying beads and attached DNA directly to the subsequent step throughout the protocol, with the relevant solution pipetted gently up and down to re-suspend beads [64]. In contrast to usual methods, which involve elution following every bead clean-up, DNA was only eluted off the beads before and after the indexing PCR – this should go some way towards reducing library loss with each elution step (e.g., a recent study of beetles up to 159 years in age found an average DNA loss of 48.7% following bead clean-up during library preparation [17]). Finally, a calculation was used to determine the number of PCR cycles to use instead of applying a blanket number, or using qPCR. This calculation was based off Table 1 in the KAPA Library Amplification Kit Technical Data Sheet (KR0408_V7,17, KAPA BioSystems; available at https://www.kapabiosystems.com/document/kapa-library-amplification-kit-tds/?dl=1), with sample concentrations determined using a Qubit dsDNA HS Assay Kit and Fluorometer (ThermoFisher Scientific).

Indexing PCRs were performed twice for each sample (12 μl volume of DNA), with a different DNA polymerase in each reaction (#KK2600, KAPA HiFi, Kapa BioSystems; and #M0530, Phusion HiFidelity, New England Biolabs, Inc.) and 9–18 PCR cycles, depending on the sample concentration following adapter ligation. Following indexing PCRs, samples were quantified and pooled with equimolarity, then hybridised to baits following a modified version of the SeqCap EZ Library SR User’s Guide (Roche). The main modification during hybridisation was use of Nimblegen SeqCap EZ Developer Reagent (Roche) in place of COT-1 DNA, as COT-1 DNA is not available for *H. armigera*.

Baits were designed by NimbleGen (Roche), with target sites encompassing ~ 1300 loci extracted from the *H. armigera* annotated genome [65]. Following hybridisation, clean-up, and amplification of the pooled library, qPCR was used to confirm the success of the capture, before sequencing was carried out on an Illumina NextSeq500 (75 bp PE) at the Biomolecular Resource Facility (Australian National University). The full wet-lab protocol is provided in Supplementary Material.

### Bioinformatics pipeline

Quality control of raw read data was performed using FastQC v.0.10.1 (http://www.bioinformatics.babraham.ac.uk/projects/fastqc/). Trimmomatic v.0.36 [66] was used to remove adapter sequences, after which trimmed reads were aligned to the *H. armigera* genome (“Harm_1.0”; GenBank assembly accession: GCA_002156985.1), which spans a total length of ~ 337 Mb, using the bwa 0.7.5a-r405 [67] mem algorithm. Duplicate reads were removed from sorted bam files using picard v.2.10.6 (http://broadinstitute.github.io/picard/), and low quality and ambiguous alignments were removed with samtools v.1.5 [68] commands: -q 20 -f 0 × 0002 -F 0 × 0004 -F 0 × 0008. Finally, bam files were indexed with samtools and evaluated with the software mapdamage v.2.0 [42] to quantify DNA damage patterns.

### Statistical analysis

The output bam files, generated above, were analysed with a variety of packages and tools, including samtools (flagstat), picard (CollectWgsMetrics, EstimateLibraryComplexity) and ngscat v.0.1 [69] to obtain various metrics of library quality. Statistical analyses (e.g., correlation, t-tests) were performed to examine the relationship between sample age and these metrics using core packages in R v.3.5.1 [70]; the R scripts are provided as Supplementary Material.

## Supplementary information

**Additional file 1.**

## Data Availability

The datasets used and/or analysed during the current study are available from the corresponding author on reasonable request.
