# Supplementary material for: Effects of sample age on data quality from targeted sequencing of museum specimens: what are we capturing in time?
Source: BMC Genomics. 2020 Feb 28;21:188. doi: 10.1186/s12864-020-6594-0 (PMC7048091; doi:10.1186/s12864-020-6594-0)
Supplement: Supplementary file 1 — Additional file 1. [file 12864_2020_6594_MOESM1_ESM.docx]

**Supplementary Material**

**Effects of sample age on data quality from targeted sequencing of museum specimens: what are we capturing in time?**

Angela McGaughran

The following protocol is broadly based on the procedures outlined in Meyer & Kircher (2010) using the standard steps for NGS library preparation (i.e., end repair, adaptor ligation and fill-in, and indexing PCR), but with several modifications due to the fragmented nature of the starting material. These modifications included: the omission of a shearing step, the incorporation of USER enzyme in the blunt-end repair step, an ‘on-beads’ clean-up protocol throughout, and the use of a calculation to determine the number of required indexing PCR cycles based on sample concentration. In addition, indexing PCRs were performed using two different DNA polymerases.

**REAGENT SOURCES**

| **Reagent** | **Supplier** | **Product Code** |
| --- | --- | --- |
| SeraMag beads | GE Healthcare Life Sciences | 45152105050250 |
| Buffer Tango (10x) | ThermoFisher Scientific | BY5 |
| dNTPs (25mM) | Life Technologies | R1122 |
| ATP (100nM) | ThermoFisher Scientific | R1441 |
| T4 polynucleotide kinase (10U/μl) | ThermoFisher Scientific | EK0032 |
| USER Enzyme | New England Biolabs | M5508 |
| T4 DNA polymerase (5U/μl) | ThermoFisher Scientific | EP0062 |
| T4 DNA ligase buffer (10x) | ThermoFisher Scientific | B69 |
| PEG-4000 (50%) | ThermoFisher Scientific | - |
| Adapter mix (100 μM) | Various* |  |
| T4 DNA ligase (5U/μl) | ThermoFisher Scientific | EL0012 |
| ThermoPol reaction buffer (10x) | Genesearch | - |
| Bst polymerase (8U/μl) | GeneSearch | M0275 |
| Kapa HiFi HotStart ReadyMix | Roche | KK260 |
| Primer IS4 | Various* |  |
| Phusion HF buffer | New England Biolabs | - |
| Phusion Taq | New England Biolabs | M0530 |
| Index Primer | Various* |  |

*Index primers, Primer IS4, and the primers in the Adapter Mix can be sourced from any company that provides primers (e.g., Sigma Aldrich). The adapter sequences can be found in Meyer & Kircher (2010) and instructions for making the Adapter master mix can be found in the supplementary material of the same article.

**LIBRARY PREPARATION PROTOCOL**

1. DNA EXTRACTION
   1. Extract DNA from specimens following your favourite extraction method.
   2. Run DNA out on a gel or other fragment analyser to determine quality, quantity and fragment sizes.
   3. If possible, avoid shearing and proceed to step 3.
2. SHEARING
   1. If necessary, shear samples to a required fragment size following standard shearing methods.
3. NON-SHEARED SAMPLES
   1. Dilute all samples into milli-Q water to your required eqimolar starting concentration and proceed to step 4.
4. PRE-LIBRARY PREP SIZE SELECTION/CLEAN-UP (DOUBLE CLEAN-UP)

Special requirements: fresh ethanol, DNA binding beads, magnet

- 1. Due to the absence of a shearing step, it may be optimal to perform a double bead clean-up as a first step to remove any large DNA fragments. In the case of sheared samples, consider starting with a standard bead clean-up as outlined below.
  2. This protocol assumes a starting volume of 30 μl of extracted DNA for non-sheared samples, and a starting volume of 90 μl for sheared samples, with the latter split into two tubes, each containing 45 μl. Over the following steps, bead volumes for sheared samples are given in **bold**, with non-sheared samples specified in normal type.
  3. Add 0.5x volume of beads (15 μl; **22.5 μl**) to sample (30 μl; **45** μl) tubes and pipette up and down to mix. Pulse spin.
  4. Let mix sit for 10 min. Make up fresh 70% EtOH during this time.
  5. Open tubes and place on magnetic stand for 4 min until liquid becomes clear.
  6. Pipette supernatant to a new tube – this removes DNA > 500 bp, but consider keeping beads for backup.
  7. Add 1.5x volume of beads (67.5 μl; **101.25** μl) to tubes, pipette up and down to mix. Pulse spin.
  8. Let mix sit for 10 min.
  9. Open tubes and place on magnetic stand for 4 min until liquid becomes clear.
  10. Pipette off supernatant, being careful not to remove beads.
  11. Leave tubes on magnet; add 150 μl of 70% EtOH. Let stand for 1 min then discard supernatant.
  12. Repeat EtOH wash, this time making sure all EtOH is removed using a 20 μl pipette.
  13. Let beads air-dry off magnet for 6 min at RT with lids open.
  14. Add 52 μl; **26** μl ddH_2_0 to each tube. Re-suspend beads by flicking and pulse spin.
  15. Let tubes sit off plate for 5 min.
  16. Open tubes on plate for 3 min.
  17. Remove 50 μl; **25** μl of eluted DNA to new tubes; combine replicates into a single tube for sheared samples. You should now have 50 μl of each sample.
  18. Proceed to step 5.

1. BLUNT-END REPAIR with USER ENZYME

Special requirements: Blunt-end repair ingredients, USER enzyme, beads, and

20% PEG

- 1. Thaw Buffer Tango, dNTPs and ATP on ice, keep enzymes in fridge until use.
  2. Make up Master Mix directly into sample tubes from step 4:

(x1) (x__)

H_2_O 4.32 _____

Buffer Tango (10x) 7 _____

dNTPs (25mM) 0.28 _____

ATP (100nM) 0.70 _____

T4 polynucleotide kinase (10U/μl) 3.5 _____

USER 3___ _____

18.8

- 1. Add 18.8 μl of master mix to each 50 μl sample.
  2. Mix samples by flicking and pulse spin, incubate for 3 h @ 37°C. At 2 hr 30, allow beads to come to room temperature and vortex until thoroughly mixed. Also bring 20% PEG to room temperature (note 20% PEG is essentially the bead solution without any beads). Complete step 6.
  3. Add 1.2 μl of T4 DNA polymerase (5U/μl) to each reaction, mix by flicking and pulse spin. Incubate @ 25°C for 15 min, followed by 5 min @ 12°C.
  4. Incubate @ 75°C for 20 min, followed by a ramp-step decrease to 12°C @ -1°/s.
  5. Proceed to step 7.

1. RETAIN-BEADS PREP (while Blunt-End Repair Step 2 is running)
   1. Add 100 μl of thawed beads to 200 μl PCR tubes. Pulse spin.
   2. Add the beads to the magnetic stand for 2 min, then remove supernatant with a fine pipette.
   3. Keep tubes.
   4. Proceed to step 7.
2. RETAIN-BEADS CLEAN-UP
   1. Add 70 μl sample from step 5g to beads from step 6d and vortex to mix.
   2. Add 2x volume of 20% PEG (140 μl) to tubes and pipette up and down to mix. Pulse spin.
   3. Let mix sit for 10 min. Make up fresh 70% EtOH during this time.
   4. Open tubes and place on magnetic stand for 4 min until liquid becomes clear.
   5. Pipette off supernatant, being careful not to remove beads.
   6. Leave tubes on magnet; add 150 μl of 70% EtOH. Let stand for 1 min then discard supernatant.
   7. Repeat EtOH wash, this time making sure all EtOH is removed using a 20 μl pipette.
   8. Let beads air-dry for 6 min at RT with lids open.
   9. Proceed to step 8 immediately, without eluting.
3. ADAPTOR LIGATION

Special requirements: Adapter ligation ingredients

- 1. Thaw T4 DNA ligase buffer, PEG-4000 and adaptor mix on ice, can vortex PEG-4000 if cloudy.
  2. Make up Master Mix on ice:

(x1) (x__)

H20 17.4 _____

T4 DNA ligase buffer (10x) 6 _____

PEG-4000 (50%) 6 _____

Adapter mix (100 μM) 0.3 _____

T4 DNA ligase (5U/μl) 0.3 _____

30

- 1. Add 30 μl master mix to samples, mix well by pipetting and pulse spin.
  2. Incubate for 30 min @ 22°C.
  3. Proceed immediately to step 9.

1. RETAIN-BEADS CLEAN-UP

Special requirements: fresh ethanol, 20% PEG, magnet

- 1. Add 2x volume of 20% PEG (60 μl) to tubes and pipette up and down to mix. Pulse spin.
  2. Let mix sit for 10 min. Make up fresh 70% EtOH during this time.
  3. Open tubes and place on magnetic stand for 4 min until liquid becomes clear.
  4. Pipette off supernatant, being careful not to remove beads.
  5. Leave tubes on magnet; add 150 μl of 70% EtOH. Let stand for 1 min then discard supernatant.
  6. Repeat EtOH wash, this time making sure all EtOH is removed using a 20 μl pipette.
  7. Let beads air-dry for 6 min at RT with lids open.
  8. Proceed to step 10 immediately, without eluting.

1. ADAPTOR FILL-IN

Special requirements: Adapter fill-in ingredients

- 1. Make up Master Mix on ice:

(x1) (x__)

H20 21.2 _____

ThermoPol reaction buffer (10x) 6 _____

dNTPs (25 mM) 0.6 _____

Bst polymerase (8U/μl) 2.2 _____

30

- 1. Add 30 μl of master mix to sample tubes, mix by pipetting and pulse spin.
  2. Incubate for 20 min @ 37°C.
  3. Proceed immediately to step 11.

1. RETAIN-BEADS CLEAN-UP

Special requirements: fresh ethanol, 20% PEG, magnet

- 1. Add 2x volume of 20% PEG (60 μl) to tubes and pipette up and down to mix. Pulse spin.
  2. Let mix sit for 10 min. Make up fresh 70% EtOH during this time.
  3. Open tubes and place on magnetic stand for 4 min until liquid becomes clear.
  4. Pipette off supernatant, being careful not to remove beads.
  5. Leave tubes on magnet; add 150 μl of 70% EtOH. Let stand for 1 min then discard supernatant.
  6. Repeat EtOH wash, this time making sure all EtOH is removed using a 20 μl pipette.
  7. Let beads air-dry for 6 min at RT with lids open.
  8. Elute the samples by adding 28 μl of milliQ-H_2_0, vortex and pulse spin.
  9. Let tubes sit off plate for 5 min.
  10. Open tubes and place on plate for 3 min.
  11. Remove 26 μl of sample to new tube.
  12. Quantify each sample to determine number of cycles for Indexing PCR, then freeze samples or proceed to step 12.

1. INDEXING-PCR

Special requirements: indexing PCR ingredients

- 1. Determine the number of PCR cycles to use for each sample, based on its concentration, as follows (calculation based on Table 1 in the KAPA Library Amplification Kit Technical Data Sheet (KR0408_V7,17, KAPA BioSystems; available at https://www.kapabiosystems.com/document/kapa-library-amplification-kit-tds/?dl=1):

| **Input DNA** | **No. of cycles** |
| --- | --- |
| 1 μg | 6 - 7 |
| 500 ng | 7 - 8 |
| 250 ng | 8 - 9 |
| 100 ng | 9 - 11 |
| 50 ng | 12 - 14 |
| 25 ng | 13 - 15 |
| 10 ng | 14 - 16 |
| 5 ng | 16 - 18 |
| 1 ng | 18 - 20 |

- 1. Make up Kapa Master Mix on ice:

(x1) (x__)

Kapa HiFi HotStart ReadyMix 12.5 ____

Primer IS4 1 ____

H20 4.5 ____

18

Index Primer [1-56] 2

DNA 12

32

- 1. Run PCR (Kapa) with 6-20 cycles of steps ii - iv. Note, these cycling conditions are optimised for the KAPA enzyme and may need adjusting:
     1. 98°C 45 s
     2. 98°C 15 s
     3. 60°C 30 s
     4. 72°C 30 s
     5. 72°C 1 min
  2. Make up Phusion Master Mix:

(x1) (x__)

H20 13.05 ____

Phusion HF buffer 5 ____

dNTPs (25 mM) 0.2 ____

Primer IS4 0.5 ____

Phusion Taq 0.25 ____

19

Index Primer [1-56] 1

DNA 12

32

- 1. Run PCR (Phusion) with *x* cycles of steps ii - iv, but note, these cycling conditions are optimised for the Phusion enzyme and may need adjusting
     1. 98°C 30 s
     2. 98°C 10 s
     3. 60°C 20 s
     4. 72°C 20 s
     5. 72°C 10 min
  2. OPTIONAL: Run out an aliquot of amplified DNA on a gel or fragment analyser to confirm indexing PCRs worked.
  3. Proceed to step 13.

1. RETAIN-BEADS CLEAN-UP

Special requirements: fresh ethanol, beads, magnet

- 1. Add 2x volume of FRESH beads (64 μl) to tubes and pipette up and down to mix. Pulse spin.
  2. Let mix sit for 15 min. Make up fresh 70% EtOH during this time.
  3. Open tubes and place on magnetic stand for 4 min until liquid becomes clear.
  4. Pipette off supernatant, being careful not to remove beads. OPTIONAL: Keep supernatant as backup.
  5. Leave tubes on magnet; add 150 μl of 70% EtOH. Let stand for 1 min then discard supernatant.
  6. Repeat EtOH wash, this time making sure all EtOH is removed using a 20 μl pipette.
  7. Let beads air-dry for 6 min at RT with lids open.
  8. Elute the samples by adding 25 μl of milliQ-H_2_0, flick and pulse spin.
  9. Let tubes sit off plate for 5 min.
  10. Open tubes and place on plate for 3 min.
  11. Remove 23 μl of sample to new tube.
  12. OPTIONAL: Run out clean amplified DNA on a gel or fragment analyser to confirm sample is retained following clean-up.
  13. Combine cleaned KAPA and Phusion PCR products into a single tube.
  14. Proceed to step 14.

1. QUANTIFICATION and POOLING
   1. Quantify DNA in each sample and pool to a final library concentration as specified in your hybridisation protocol.
   2. Proceed to step 15.
2. HYBRIDISATION
   1. Complete the hybridisation steps following the recommended protocol for your designed targets.
3. CLEAN and QC THE AMPLIFIED CAPTURED DNA
4. SEND FOR SEQUENCING

References

Meyer, M., & Kircher, M. (2010). Illumina Sequencing Library Preparation for Highly Multiplexed Target Capture and Sequencing. *Cold Spring Harbor Protocols*, *2010*(6), db.prot5448 – pdb.prot5448.
